# Risk factors of adjacent vertebral collapse after percutaneous vertebroplasty for osteoporotic vertebral fracture in postmenopausal women

**DOI:** 10.1186/s12891-016-0887-0

**Published:** 2016-01-12

**Authors:** Kenji Takahara, Mikio Kamimura, Hideki Moriya, Ryohei Ashizawa, Tsuyoshi Koike, Yohei Hidai, Shota Ikegami, Yukio Nakamura, Hiroyuki Kato

**Affiliations:** Department of Orthopedic Surgery, Ina Central Hospital, Ina, 396-8555 Japan; Center of Osteoporosis and Spinal Disorders: Kamimura Orthopaedic Clinic, Matsumoto, 399-0021 Japan; Department of Orthopaedic Surgery, Shinshu University School of Medicine, Asahi 3-1-1, Matsumoto, 390-8621 Japan

**Keywords:** Adjacent vertebral collapse, Osteoporotic vertebral fracture, Percutaneous vertebroplasty, Risk factors

## Abstract

**Background:**

Recently percutaneous vertebroplasty (PVP) was frequently performed for treatment of osteoporotic vertebral fractures (VFs). It is widely accepted that new compression fractures tend to occur adjacent to the vertebral bodies, typically within a month after PVP. To determine the risk factors among several potential predictors for *de novo* VFs following PVP in patients with osteoporosis.

**Methods:**

We retrospectively screened the clinical results of 88 patients who had been treated by PVP. Fifteen cases were excluded due to non-union. Of the remaining 73 patients, 19 (26.0 %) later returned with pain due to a new vertebral compression fracture. One patient with a non-adjacent fracture and 2 patients with adjacent factures occurring 3 months later were excluded from the study. The 9 male patients were excluded to avoid gender bias. Ultimately, we divided the 61 remaining postmenopausal female patients (mean age: 78.9 years) into the collapse group (14 patients) who had experienced adjacent vertebral collapse after PVP and the non-collapse group (47 patients) who had not. Logistic regression analysis was performed to identify the risk factors for new VFs after PVP.

**Results:**

All 14 cases of adjacent VF occurred within the first month after surgery. The collapse group had significantly advanced age, higher urinary N-terminal cross-linking telopeptide of type I collagen, and lower lumbar and hip bone mineral density (BMD) scores as compared with the non-collapse group. The odds ratios for age, lumbar, total hip, femoral neck, and trochanteric BMD were 4.5, 8.2, 4.5, 7.2, and 9.6, respectively. Positive likelihood ratios suggested that age more than 85 years, lumbar BMD less than 0.700 [−2.6SD], total hip BMD less than 0.700 [−1.8SD], neck BMD less than 0.600 [−2.1], and trochanter BMD less than 0.600 conferred an elevated risk of adjacent VF.

**Conclusions:**

Our study revealed that advanced age and decreased lumbar and hip BMD scores most strongly indicated a risk of adjacent VF following PVP.

## Background

The incidence of osteoporotic fractures is increasing yearly in Japan. As these injuries greatly diminish quality of life (QOL) and activities of daily living in the elderly, methods of preventing osteoporosis and osteoporotic fractures are urgently required [1, 2]. Vertebral fractures (VFs) have also been increasing and are now considered to be common disorders that are especially problematic in the elderly. In particular, osteoporotic VFs frequently cause persistent back pain, which significantly impairs mobility and QOL [3].

In 1987, Galibert first described a procedure employing the percutaneous injection of polymethylmethacrylate (PMMA) into the vertebral body in patients with vertebral angioma [4]. Soon afterwards, indications for percutaneous vertebroplasty (PVP) spread widely to include osteoporotic vertebral compression fracture. PVP provides increased strength and pain relief in vertebrae weakened by osteoporosis [4]. In their review of 1000 consecutively treated vertebral compression fractures, Layton et al. found that this technique had a high success rate and low complication rate for cases of painful spinal compression fractures [5].

Of the reported complications associated with PVP, the most frequent are VFs [6]; patients successfully treated by vertebroplasty often return with new pain caused by a *de novo* vertebral body fracture. Therefore, it is considered important to investigate the risk factors for such compression fractures, although many have been controversial because of limited data and a lack of uniformity in measurement and evaluation [7].

It is widely accepted that new compression fractures tend to occur adjacent to the vertebral bodies that were initially treated [8], typically within a month after surgery [9]. Therefore, with respect to PVP using PMMA, it is paramount to mitigate the risk and prevent the occurrence of adjacent vertebral compression fractures soon after surgery. There have been several reports on the risk of fracture following operations for VF [8, 10]. However, since these studies were over differing follow-up periods and did not include such factors as bone turnover markers, we focused exclusively on adjacent VFs manifesting within a month after surgery and analyzed patient characteristics, bone marker values, and bone mineral density (BMD) scores to identify risk factors associated with clinical outcome.

## Methods

We retrospectively screened the case data of 88 consecutive patients with osteoporotic vertebral compression fracture who were treated at our institution between July 2004 and July 2012. Seventy-three cases of fresh fracture were treated by PVP, while 15 cases were excluded due to non-union. There were no patients with multiple fractures during the study period. The indication for PVP was incidental VF, regardless of radiographic vertebral collapse, causing significant back pain that could not be relieved by conservative measures, such as non-steroidal anti-inflammatory drug (NSAID) treatment. All recruited patients could not move without unbearable back pain. The surgeries were performed between 1 and 16 days (mean: 7.4 days) after pain onset. Contraindications included medical conditions such as bleeding disorder and a lack of definable vertebral collapse. No patient had received prior treatment with bisphosphonates (BPs) or any other osteoporosis medication.

Surgery was performed for 73 cases of VF, and *de novo* vertebral compression fractures were witnessed in 19 cases within 1 year. Among them, a non-adjacent vertebral compression fracture occurred in 1 patient within a month after surgery, and 2 patients returned with adjacent vertebral compression fractures 3 months later. These subjects were excluded from the study. Thus, we encountered16 patients with osteoporotic adjacent vertebral compression fracture occurring within a month after surgery. After initial screening, 70 patients (9 men and 61 postmenopausal women; mean age: 77.2 years, range: 56–96 years) who underwent elective PVP using PMMA were considered for this study.

During PVP, there were 7 cases with cement leakage, all of whom were female. PMMA leakage into soft tissues and intradiscal tissues was observed in 4 and 3 patients, respectively. Whereas the former patients had no adjacent VF, all 3 of the latter patients belonged to the collapse group in this study. Furthermore, both leakage subgroups had significantly lower BMD than the remaining cases (intradiscal L2–4 BMD: 0.521, *P* = 0.03 and soft tissue L2–4 BMD: 0.601, *P* < 0.05).

The male cases were then excluded from statistical analyses to avoid gender bias. Among the remaining 61 female patients (mean age: 78.9 ± 7.8 years), 14 experienced osteoporotic adjacent vertebral compression fracture occurring within a month after surgery.

Before PVP, all patients were evaluated using plain radiographs and magnetic resonance imaging (MRI) to confirm fracture severity and location. The diagnosis of VF by MRI was made when T1-weighted (T1W) images showed low intensity signals and short τ inversion recovery (STIR) revealed high intensity signals in the involved vertebra in the sagittal plane. Two nationally certified spinal surgeons and an experienced radiologist examined all plain radiographs and MRI scans in this study. The distribution of the fractured vertebrae was as follows: T11, 14; T12, 23; L1, 16; L2, 7; and L4, 1 (Table 1). Pain was evaluated using a visual analog scale (VAS).

**Table 1:** Fracture and adjacent fracture levels

Fracture level	N	Adjacent new fracture n (%)	Level (n)
T11	14	5 (35.8 %)	T10 (3), T12 (2)
T12	23	5 (21.7 %)	T11 (4), L1 (1)
L1	16	3 (18.8 %)	T12 (3)
L2	7	1 (14.3 %)	L1 (1)
L4	1		

PVP was performed within several days of hospitalization for all cases. The patients were first placed in a prone position on the operating table. Two single-plane mobile C-arms were positioned to confirm the anterior-posterior and lateral views of the fractured vertebra. Local anesthesia was administered topically and in the subcutaneous tissues, muscular tissues, and periosteum of the targeted pedicle. An 11-gauge bone-puncturing needle was used. PMMA was bilaterally injected into the fractured vertebral body with a 1-mL syringe until the cement reached the posterior one-fourth of the body. Walking while wearing a corset was permitted from the following day, and patients were discharged within 7 days. The patients were monitored using plain radiography at 1, 3, 6, and 12 months postoperatively. Patients who experienced new pain during the follow-up period were brought back for additional MRI examination of the thoracic and lumbar spine. BP treatment was commenced for all patients after PVP.

*De novo* adjacent vertebral compression fractures were confirmed by plain radiographs and MRI within a month of surgery in 14 patients (i.e., the collapse group). We compared the clinical and laboratory findings of the collapse group with those of the 47 patients without adjacent vertebral collapse (i.e., the non-collapse group). VAS scores for pain taken before PVP and at 1 day, 1 month, and 3 months of follow-up were also compared between the groups, as were bone turnover marker values and BMD scores.

The day of admission to our facilities was that of pain onset for almost all cases. Blood and urinary tests were performed on the morning following hospitalization for all subjects. We measured serum bone alkaline phosphatase (BAP) and urinary N-terminal cross-linking telopeptide of type I collagen (NTX) as markers of bone formation and resorption, respectively. NTX levels were measured with an enzyme-linked immunosorbent assay (ELISA) (OSTEOMARK; Osteox International, Seattle, WA) by SRL Inc. (Tokyo, Japan). On the first admission day, BMD scores of the lumbar spine (L2–4) and unilateral hip (total hip and trochanteric and femoral neck sites) were determined using dual X-ray absorptiometry (DXA) (Hologic, Waltham, MA, USA). Results were expressed as the mean and standard deviation (SD) of data whenever applicable. The change of VAS in each group was assessed with paired t-test with Bonferroni correction. Also, we performed Student’s t-test with respect to the compared values of VAS between 2 groups. Differences between the groups for bone turnover marker values and BMD scores were assessed using Welch’s t-test. Logistic regression analysis was performed to obtain the risk ratios (odds ratio; OR).and determine the predictors of vertebral collapse after PVP. The changed OR was shown by the changes of 1 SD in each parameter, We next calculated the sensitivity, specificity, positive likelihood ratio (LR+), and negative likelihood ratio (LR-) of promising prognostic factors. These parameters were determined to be useful in predicting adjacent VF if the LR+ was greater than 5.0 and the LR- was less than 0.2. Differences were considered to be statistically significant when P values were less than 0.05. All analyses were performed using SPSS software for Windows version 17.0 (SPSS, Chicago, IL, USA).

This study has been performed in accordance with the ethical standards laid down in an appropriate version of the revised 2014 Declaration of Helsinki. All patients provided informed written consent prior to their inclusion in the investigation. This study has been approved by the ethics committee at Ina Central Hospital.

## Results

*De novo* fractures were identified in 14 of 61 osteoporotic female patients (23.0 %) within the first month of the follow-up period. The distribution of affected vertebrae is listed in Table 1. The first and adjacent VFs occurred frequently at the thoracolumbar junction. Eleven of 14 fractures (78.6 %) occurred immediately above the original VF after PVP, while 3 of 14 fractures (21.4 %) were seen just below. This difference was significant (*P* = 0.015, Fisher’s exact test).

After PVP, pain relief and improvement in mobility was obtained in the short term. From postoperative 1 day to final follow-up, VAS scores significantly showed lower values compared with preoperative scores in both groups. However, at 1 month of follow-up, the mean VAS score in the collapse group (6.8 ± 1.2) showed significantly higher values in comparison with those in the non-collapse group (2.5 ± 1.0) (*P* < 0.001), likely because of the occurrence of a new adjacent VF (Table 2). The mean VAS pain score in the collapse group later showed lower values to 3.3 ± 0.8 at final follow-up to again become similar to that in the non-collapse group (2.6 ± 1.2). However, the VAS score in the collapse group showed higher values than those in the non-collapse group (*P* = 0.015). In both group, at final follow-up VAS score significantly improved compared with preoperative scores.

**Table 2:** Mean VAS pain scores in the non-collapse and collapse groupsᅟ

	Non-collapse group (*n* = 47)	Collapse group (*n* = 14)	*P*-value between the 2 groups
Initial	9.7 ± 0.5	9.6 ± 0.8	*P* = 0.600
After 1 day	1.5 ± 0.7*	1.8 ± 1.0*	*P* = 0.356
After 1 month	2.5 ± 1.0*	6.8 ± 1.2*	*P* < 0.001
Final follow-up	2.6 ± 1.2*	3.3 ± 0.8*	*P* = 0.015

Parameters were compared between the 2 groups using the Student’s t-test

*Significant difference. Compared with pre-operative VAS scores

Demographic comparisons between the 2 groups are found in Table 3. Average age was significantly higher in the collapse group (*P* < 0.001). Height, weight, and body mass index (BMI) were not remarkably different. The level of urinary NTX in the collapse group was significantly higher than that in the non-collapse group (*P* = 0.036) (Table 3). However, there was no significant difference in BAP levels. In the collapse group, lumbar (*P* < 0.001), total hip (*P* = 0.001), femoral neck (*P* < 0.001), and trochanter BMD (*P* < 0.001) scores were all significantly lower than those in the non-collapse group (Table 3). The results excluding the 3 cases with cement leakage into discs were comparable (data not shown).

**Table 3:** Patient characteristics and overall findings in the non-collapse and collapse groups

	Non-collapse group *n* = 47	Collapse group *n* = 14	*P* value
Height (cm)	144.5 ± 3.8	142.6 ± 3.0	0.066
Weight (kg)	43.9 ± 5.4	42.0 ± 3.7	0.150
BMI (kg/m^2^)	21.0 ± 2.1	20.6 ± 1.5	0.497
Age (years)	77.0 ± 7.5	85.2 ± 5.8	<0.001*
BAP (U/L)	38.7 ± 16.3	35.1 ± 11.1	0.356
NTX (nmol BCE/mmol Cr)	60.8 ± 20.9	72.1 ± 15.6	0.036*
L2–4 BMD (g/cm^2^)	0.776 ± 0.101	0.620 ± 0.084	<0.001*
Total BMD (g/cm^2^)	0.814 ± 0.079	0.699 ± 0.103	0.001*
Neck BMD (g/cm^2^)	0.759 ± 0.086	0.618 ± 0.103	<0.001*
Troch BMD (g/cm^2^)	0.714 ± 0.096	0.574 ± 0.090	<0.001*

*Significant difference

Logistic regression was used to determine the risk ratios of variables possibly influencing vertebral collapse after PVP (Table 4). Five risk factors (+1 SD for age, −1 SD for L2–4 BMD, and −1 SD each for total, neck, and trochanter BMD) were found to be significant in a binary logistic model (*P* < 0.05). In particular, the OR of total hip, femoral neck, and trochanteric BMD were 4.5, 7.2, and 9.6, respectively.

**Table 4:** Odds ratio and 95 % CI for risk of adjacent vertebral fracture

Risk factor	Odds ratio	95 % CI	*P* value
−1SD of BMI	1.2	0.6–2.3	0.562
+1SD of age	4.5	1.9–13.0	0.001
+1SD of NTX	1.9	0.9–4.1	0.073
+1SD of BAP	0.7	0.3–1.4	0.442
−1SD of L2–4 BMD	8.2	2.9–34.4	<0.001
−1SD of total BMD	4.5	2.0–13.3	0.001
−1SD of neck BMD	7.2	2.6–28.8	<0.001
−1SD of troch BMD	9.6	3.0–51.2	0.001

*Abbreviations*: *SD* standard deviation, *CI* confidence intervals

Table 5 shows the sensitivity (%), specificity (%), LR+, and LR- of the above 5 risk factors implicated with *de novo* adjacent vertebral collapse, as follows:

**Table 5:** Sensitivity, specificity, and cut-off values of factors predictive of adjacent vertebral fracture

Parameter	Sensitivity (%)	Specificity (%)	LR+	LR-
Age (years)				
≥75	100	30	1.42	0.00
≥ 80	79	70	2.63	0.30
≥ 85	57	83	3.35	0.51
85 and over	21	96	5.03	0.82
L2–4 BMD (g/cm^2^)				
< 0.600 [−3.5SD]	29	94	4.47	0.76
< 0.700 [−2.6SD]	86	83	5.03	0.17
< 0.800 [−1.8SD]	100	34	1.51	0.00
< 0.900 [−0.9SD]	100	11	1.11	0.00
Total BMD (g/cm^2^)				
< 0.600 [−2.8SD]	29	100	∞	0.71
< 0.700 [−1.8SD]	43	98	20.1	0.58
< 0.800 [−0.8SD]	93	53	1.98	0.13
< 0.900 [+0.3SD]	100	9	1.09	0.00
Neck BMD (g/cm^2^)				
< 0.600 [−2.1SD]	36	98	16.78	0.65
< 0.700 [−1.0SD]	71	74	2.79	0.38
< 0.800 [+0.1SD]	100	28	1.38	0.00
< 0.900 [+1.2SD]	100	6	1.06	0.00
Troch BMD (g/cm^2^)				
<0.600 [-]	50	96	11.75	0.52
<0.700 [-]	100	51	2.04	0.00
<0.800 [-]	100	21	1.27	0.00
<0.900 [-]	100	4	1.04	0.00

*Abbreviations*: *BMD* bone mineral density, *LR+* positive likelihood ratio, *LR-* negative likelihood ratio. SD values were calculated according to the Japanese Orthopaedic Diagnostic Guidelines using cut-off values based on Japanese patients. The SD of trochanteric BMD could not be calculated since there are no established values in Japan

∞: infinity

Age: LR+ was more than 5.0 until 85 years of age and then 5.03 for 85 years and older. LR- was 0.20 or more for age 75 and over but was less than 0.20 until age 75. These results suggest that vertebroplasty is suitable for patients who are less than 75 years old since the risk of adjacent vertebral collapse appears to be low. In contrast, PVP may not be ideal for patients over 85 due to a high risk of complications.

Lumbar spine BMD: LR+ was above 5.0 when L2–4 BMD was less than 0.700 (−2.6SD), implying a high risk of adjacent VF. LR- was below 0.20 when BMD was 0.700 or more (−2.6SD), which indicated a low risk of complications.

Total hip BMD: LR+ was above 5.0 when hip BMD was less than 0.700 (−1.8SD), suggesting a high risk of adjacent VF. LR- was below 0.20 when BMD was 0.800 (-0.8SD) or more, inferring a low risk of adjacent VF.

Femoral neck BMD: LR+ was above 5.0 when femoral neck BMD was less than 0.600 (−2.1SD), indicating a high risk of adjacent VF. LR- was below 0.20 when BMD was 0.800 or more (+0.1SD), suggesting a low risk of complications.

Trochanteric BMD: LR+ was above 5.0 when trochanteric BMD was less than 0.600, which indicated a high risk of adjacent VF. LR- was below 0.20 when BMD was 0.700 or more, which was suggestive of a low risk of adjacent VF.

## Discussion

In our evaluation of predictive factors for *de novo* adjacent compression fractures following PVP, we uncovered statistically significant relationships between an increased risk of complications and the variables of age and BMD scores. Interestingly, decreased lumbar and hip BMD scores both indicated a high risk of adjacent VFs. Based on our LR results, patients 85 years of age or older with low BMD scores do not represent good surgical candidates for PVP. In contrast, those less than 75 years old with higher BMD scores have a relatively low risk of fracture, suggesting that these individuals are suitable for PVP intervention.

Several authors have reported that the mechanism for new fractures is a combination of the usually osteoporotic underlying condition and the hard cement injected into the vertebral body [11–14]. Trout et al. described that vertebral bodies adjacent to those treated with vertebroplasty had greater than 4 times the risk of fracture than non-adjacent vertebrae. The authors also showed that new-onset adjacent-level fractures after PVP occurred significantly sooner than non-adjacent ones [8]. Moreover, Uppin et al. revealed that many adjacent VFs occurred within a month after PVP [15]. In our preliminary subject screening, 18 of 19 new fractures (94.7 %) had affected adjacent vertebrae, 17 of 19 fractures (89.5 %) had occurred within a month after surgery, and 16 of 19 fractures (84 %) were both adjacent and manifested within a month. Indeed, it appears critical to prevent adjacent VFs in the month following PVP treatment. We therefore focused on these parameters when analyzing clinical factors, bone marker values, and BMD scores in the present study.

Adjacent fractures occurred more significantly in the upper vertebrae. In general, kyphosis occurs in thoraco-lumbar fractures due to the collapse of the anterio-superior vertebrae. When the regions are surgically repositioned, defects in the upper vertebrae may become present and cement might enter the fronto-tepomoral regions. Nagaraja et al. recently showed that vertebroplasty alters the biomechanics of the spine, which increases compression on adjacent vertebral bodies and intervertebral discs, especially in severely osteoporotic women [16]. Furthermore, in vitro studies have revealed that cement-filled bone is 36 times stronger than spinal cancellous bone [17]. Therefore, it is plausible that more fractures occur in the upper vertebrae that make contact with the solid cement after PVP.

Several risk factors have been described with respect to adjacent VFs following vertebroplasty [7, 18–20]. Zhang et al. identified the significance of low lumbar BMD, low BMI, and intradiscal cement leakage in *de novo* fractures [7], while Ryo et al. implicated osteoporosis and intervertebral discal cement leakage [20]. Based on these reports, the strongest prognostic clinical factors appear to be lumbar BMD-related conditions and cement leakage.

Smaller volumes of cement have been shown to decrease the risk of newly forming VFs while maintaining sufficient stability [16]. In the present series, PMMA was injected into the vertebral body until the cement reached the posterior one-fourth of the body, as indicated by another group [17], or until significant leakage occurred. We had anticipated that the amount of leakage would be relatively small due to our careful adjustment of the amount and distribution of cement administration. However, 4 and 3 patients experienced soft tissue and intradiscal leakage of PMMA, respectfully. The 7 patients in whom leakage was found had significantly lower BMD scores than the remainder of the cohort. This suggests that diminished BMD is also a serious risk factor with respect to cement leakage.

Syed et al. revealed that subsequent adjacent and non-adjacent fractures after PVP occurred at roughly equal frequencies in disc extravasation and non-disc extravasation groups. [21]. However, considerable evidence supports that cement leakage is a primary risk factor for new vertebral compression fractures [7, 10, 19]. As the 3 patients who experienced leakage into the disc space were all in the collapse group, we suspected that leakage of cement into this area influenced adjacent VF onset. The BMD of the 4 cases of cement leakage into soft tissue was also significantly lower than that of the remaining cases. However, such leakage did not lead to adjacent VF. Thus, it appears that cement leakage itself does not lead to VFs; rather, increased disc stiffness after leakage may have increased the risk of adjacent compression fracture.

The cumulative incidence of VF over 10 years of follow-up was shown to be between 5.1 and 22.2 % by Yoshimura et al. [22]. Zhang et al. also reported that age was a prominent risk factor in the occurrence of fractures. We witnessed that the collapse group was significantly older than the non-collapse group (Table 3). Furthermore, the OR of adjacent fractures when age was increased by 1SD was 4.5 (*P* = 0.001). Although age is a well known risk factor for fractures, there have been few reports on its relationship with PVP. Trout et al. stated that 186 new VFs occurred in 86 of 432 patients (19.9 %). In this study, 19 of 73 patients (26.0 %) experienced *de novo* VFs, which was comparably higher. One possible reason for this discrepancy is age; the median age was 75.2 years in Trout’s report [8] versus 77.2 years in ours. Nevertheless, such findings confirm that age plays an important role in the frequency of subsequent VFs. In the present study, the risk of fracture in women was high at an age of more than 85 years and low for an age of less than 75. Therefore, an increased fracture risk should be expected in elderly women aged 85 years or older.

It is widely known that fracture risk rises in patients with high bone turnover [23]. Our findings revealed that the level of urinary NTX in the collapse group was significantly higher than that in the non-collapse group (Table 3). However, there was no significant difference by logistic regression analysis (*P* = 0.073). Serum BAP levels were not apparently related to the incidence of new fractures. These results resembled those of Komemushi et al., who reported that a combination of high levels of bone resorption markers and normal levels of bone formation markers may be associated with an increased risk of *de novo* fractures after PVP [24]. Urinary NTX values in our previously reported cohort of osteoporotic outpatients was 62.2 on average, which had risen to 76.7 on average in patients over 80 years of age [25]. Taken together, it is conceivable that in accordance with aging, the elevated values of urinary NTX may be related to fracture risk.

According to the International Society for Clinical Densitometry and International Osteoporosis Foundation, one of the key clinical risk factors for osteoporosis is diminished hip BMD [26]. In the present study, the strongest risk factors for new VFs were lumbar as well as hip BMD. Trochanteric BMD showed the highest statistical correlation among hip BMD scores (Odds ratio: 9.6). It is currently unknown why trochanteric BMD might prognosticate VFs, although this may be due to the fact that the trochanteric region involves far more cancellous bone than cortical bone. The high risk values of BMD based on LR+ were less than −2.6SD for lumbar, less than −1.8SD for total hip, and less than −2.1SD for femoral neck. The low risk values of BMD based on LR- were −1.8SD for lumbar, less than −0.8SD for total hip, and less than +0.1SD for femoral neck. These results indicate that an important factor in the prediction of adjacent VFs may be hip BMD as much as lumbar BMD. Although correlations exist between spine and hip BMD, they are insufficient to assess the counterpart’s BMD value [27]. Therefore, not only vertebral BMD, but also hip BMD and especially trochanteric BMD, should be assessed before vertebroplasty.

The main limitations of this study are its retrospective design and relatively small sample size. However, as we performed strict statistical analyses to compensate for the latter shortcoming, we consider our findings to be applicable in the treatment of spinal fractures using PVP.

The merit of vertebroplasty is still controversial. Lindsay et al. reported that while some clinicians felt vertebroplasty was an effective procedure in select patients, others believed that other treatment modalities for osteoporotic compression fractures were preferable to surgery [28]. We earlier treated such cases conservatively with rest. Patients required 3 weeks on average to regain mobility following a reduction in pain [29]. Meanwhile, PVP has few complications and can improve pain very quickly, and thus represents an effective, low invasive option. However, the occurrence of adjacent fractures remains a pressing issue. We propose that not only technical surgical improvements, such as the amount of injected cement, but also preoperative risk evaluation for new adjacent fractures along with postoperative treatment with teriparatide and other biologics, may enhance the merits of PVP.

## Conclusion

Not only lumbar, but also hip, BMD scores strongly reflect a risk of adjacent VF following PVP. As patients over 85 years of age with low BMD scores showed the highest risk of the fracture, PVP may be contraindicated in such individuals. In contrast, we uncovered a good surgical indication in patients less than 75 years old and having high BMD scores.
